# Perceived sidedness and correlation to vertical movement asymmetries in young warmblood horses

**DOI:** 10.1371/journal.pone.0288043

**Published:** 2023-07-07

**Authors:** Anna Leclercq, Johan Lundblad, Emma Persson-Sjodin, Katrina Ask, Ebba Zetterberg, Elin Hernlund, Pia Haubro Andersen, Marie Rhodin

**Affiliations:** Department of Anatomy, Physiology and Biochemistry, Swedish University of Agricultural Sciences, Uppsala, Sweden; Sul Ross State University, UNITED STATES

## Abstract

The prevalence of vertical asymmetries is high in “owner-sound” warmblood riding horses, however the origin of these asymmetries is unknown. This study investigated correlations between vertical asymmetries and motor laterality. Young warmblood riding horses (N = 65), perceived as free from lameness were evaluated on three visits, each comprising objective gait analysis (inertial measurement units system) and a rider questionnaire on perceived sidedness of the horse. A subgroup (N = 40) of horses were also subjected to a forelimb protraction preference test intended as an assessment of motor laterality. We hypothesized associations between vertical asymmetry and motor laterality as well as rider-perceived sidedness. Vertical asymmetry was quantified as trial means of the stride-by-stride difference between the vertical displacement minima and maxima of the head (HDmin, HDmax) and pelvis (PDmin, PDmax). Laterality indexes, based on counts of which limb was protracted, and binomial tests were used to draw conclusions from the preference tests. In the three visits, 60–70% of horses exhibited vertical asymmetries exceeding clinically used thresholds for ≥1 parameter, and 22% of horses exhibited a side preference in the preference test as judged by binomial tests. Linear mixed models identified a weak but statistically significant correlation between perceived hindlimb weakness and higher PDmin values attributable to either of the hindlimbs (p = 0.023). No other statistically significant correlations to vertical asymmetry were seen for any of the questionnaire answers tested. Tests of correlation between the absolute values of laterality index and asymmetry parameters (HDmin, HDmax, PDmin, PDmax) identified a weak correlation (p = 0.049) with PDmax, but when accounting for the direction of asymmetry and motor laterality, no correlations were seen for either of the asymmetry parameters. No convincing evidence of associations between vertical asymmetries and motor laterality were seen and further studies investigating motor laterality and the origin of vertical asymmetries are needed.

## Introduction

Systems for objective gait analysis in horses are gaining popularity in clinical settings as they can help veterinarians overcome interpretation bias and limitations posed by the human eye [1–4], as well as provide repeatable results during lameness examinations [5]. However, it has been shown that large proportions of “owner-sound” horses exhibit vertical movement asymmetries above thresholds used in practice to define whether lameness is present or not [6–9]. Earlier studies have reported occurences of 57% [7], 60–67% [8] and 73% [9], where the vertical asymmetries may be similar in magnitude to those seen in horses evaluated and treated for lameness [10].

It is currently unknown whether these vertical asymmetries in owner-sound horses are caused by painful processes, which would imply animal welfare issues, or if there are other explanations. Motor laterality has been discussed as a potential contributing factor to the high prevalence of asymmetries [7, 9]. However, potential associations have, to this date, not been explored.

The term “laterality” can be explained as “biased behavior” and is attributed to specialization of brain hemispheres. Laterality has been found to exist in several species, including horses [11–16], and can be studied on a population level as well as on an individual level. In previous research, horses have been shown to exert lateralized behavior in different situations [12–16], including tests recording the preferred forelimb to initiate movement, preferred side when rolling and preferred side when rounding an obstacle (ridden and unridden) [13]. In one study, 24% out of 17 young warmblood horses were shown to exhibit lateralized behavior when subjected to a preference test, where the protracted forelimb when feeding from a bucket at ground level was recorded for 15 observations per horse. Laterality was found to be correlated with body conformation; biased behavior was linked to smaller heads, longer limbs and uneven front hoofs [15]. Further, in a population of 106 thoroughbreds of varying age, 50% were found to prefer to protract one forelimb over the other when grazing [12].

Experienced riders have also claimed perceiving a “sidedness”, described as a “strong side” and a “weak side”, in riding horses [17]. It has been suggested that rider-perceived sidedness could possibly imply inherent motor laterality but the topic has only been scarcely researched. In the aforementioned study, rider-perceived sidedness was reported in all horses (n = 30) studied. Out of these, 25 horses reportedly exhibited a leftward sidedness, and the remaining five exhibited a rightward sidedness. This was based on the perception of riders during training, where the ability of the horses to perform different tasks, such as lateral movements and turns in both directions, was judged subjectively [17].

Hence, vertical asymmetries, rider-perceived sidedness and motor laterality have been found to be relatively common in horses when explored separately, but how (and if) they are connected is yet to be explored. Within the equestrian community, symmetry in movement patterns of horses is generally regarded as positive. Symmetrical gaits, such as the trot, are suitable for objective gait analysis in horses, as deviations from symmetry in vertical displacement can be quantified. Since motor laterality also refers to deviations from left/right symmetry, exploring potential associations between vertical asymmetries and motor laterality is of interest. Therefore, we aimed to investigate this matter by comparing vertical asymmetry data with results from a preference test intended to assess motor laterality and questionnaire responses regarding rider-perceived sidedness. We hypothesized that inherent motor laterality and rider-perceived sidedness would be associated with the presence of vertical asymmetries in young warmblood horses.

## Materials and methods

### Outline

Young warmblood riding horses were assessed during 3 visits, each including objective gait analysis and a questionnaire about rider perceived sidedness to be completed by the rider. A subgroup was also recruited to perform a preference test (PT) designed to assess motor laterality.

### Horses

Warmblood sport horses under the age of four, perceived as sound by their owners and not ridden in any other gaits than walk, were recruited by convenience sampling in Sweden for participation in a larger study. The horses were subjected to objective gait analysis on 9 occasions (approximately every 3^rd^ month during 2 years). For each visit, the riders completed a questionnaire regarding rider-perceived sidedness. In this study, data from measurements number 7–9 (hereafter referred to as V1-V3) is used. In cases when horses did not meet inclusion criteria regarding age (i.e. 3–5 years) for one or more of visits V1-V3, data from three consecutive earlier visits (e.g. 6–8), as close in time as possible to the visits originally intended for inclusion, were used. Inclusion criteria applied for V1-V3: age of 3–5 years and no report of lameness or orthopedic disease at time of visit. Additionally, horses had to have been sufficiently ridden, as judged by the rider, for the rider to be able to provide ≥1 answer for the questionnaire on owner-perceived laterality for at least one of V1-V3. Exclusion criteria were one or more of the following: moderate or severe distension (based on clinical orthopedic examination performed by a veterinarian at each visit) in one or more joints/associated synovial structures, visually detectable lameness ≥1 degree on a 0–5 ordinal scale during straight line trot.

A subgroup (selected based on convenience sampling) of the above-mentioned horses was also subjected to a preference test (PT) with the intention of assessing motor laterality, performed either in conjunction with one of the visits in the larger study mentioned earlier in this section, or on a later separate occasion (see below for details). For this procedure, the only criterion applied for inclusion was no report of lameness or orthopedic disease at time of visit. Thus, these horses were included even if they had passed the above-mentioned age at the time of the PT.

Written informed consent was obtained from all owners of horses participating in the study. As the animals used in the study were privately owned and no invasive procedures were carried out, no ethical permission was needed according to local legislation (SJVFS 2019:9).

### Objective gait analysis

Objective gait analysis was performed using an inertial measurement unit (IMU) system (Lameness Locator, Equinosis, St. Louis, MO, USA). The system consisted of two accelerometers (attached to the poll and between the tubera sacrale, respectively) and one gyroscope (attached dorsally on the pastern of the right forelimb). Data were transmitted wirelessly to a nearby tablet. The data collection was performed in straight line trot, and the type of surface (soft or hard) was recorded for each horse. Horses were handled by their owners or a representative. The handler was instructed to avoid disturbing the movement of the horse’s head. Differences between the two vertical displacement (local reference frame) minima and maxima for each stride were calculated by the software (Equinosis) for head and pelvis and expressed as the asymmetry parameters HDmin, HDmax, PDmin, and PDmax, where negative values indicated asymmetries attributable to the left side, while positive values indicated asymmetries attributable to the right side. Subsequently, stride-by-stride data were exported to Matlab (Release 2019a, The MathWorks Inc) where means and standard deviations (SD) for the four asymmetry parameters were calculated in custom-made scripts. Outlier removal for the head parameters was executed automatically; each stride value was compared to the average value of all strides using the Mahalanobis distance method. Strides where the parameter value exceeded the mean (for the respective parameter) with three or more standard deviations were removed. This procedure was repeated iteratively, terminating when no more outliers were found.

Horses were classified as either symmetrical or asymmetrical for each parameter based on threshold values (|6| mm for head parameters and |3| mm for pelvic parameters) which are recommended by the manufacturer (Equinosis) and clinically used. For a horse to be classed as asymmetrical on a parameter, the value had to exceed the threshold and the standard deviation had to be ≤100% of the parameter value in question (4), i.e. remaining horses were classed as symmetrical. A total asymmetry score, $TAS=\frac{\left|HDmin\right|}{2}+\frac{\left|HDmax\right|}{2}+|PDmin|+|PDmax|$, was also calculated for each horse and visit.

### Questionnaire about rider perceived sidedness

At each of V1-V3, the rider was asked to complete a questionnaire about rider-perceived sidedness. In this study, a selection of these questions deemed relevant for the purpose were used for analysis. The selected questions are presented in Table 1. For the complete questionnaire, see S1 Text.

**Table 1:** Questionnaire about rider-perceived sidedness.

Question	Preset answer options
1. Do you perceive your horse as exhibiting a sidedness? If so, grade it.	*no/mild/moderate/severe*
2. Do you perceive your horse as having a weaker hindlimb?	*no/left/right*
2b. If so, is it most noticeable as inner or outer limb?	*equally/inner/outer*
3. Which side of your horse do you perceive as the stiffest?	*none/left/right*
4. Which side of your horses’ neck do you perceive as the stiffest?	*none/left/right*
5. Do you perceive it as more difficult for your horse to perform canter strike offs in any lead?	*no/left/right*

Questionnaire consisting of six questions and preset answer options, delimited by “/”, used for analysis. The answer option “no perception” was also included for all questions.

### Preference test

The PTs were performed either in conjunction with one of the visits in the larger study mentioned in section “Horses” (5 horses), or at a later separate occasion (36 horses). In cases when it was performed after the conclusion of the larger study, an additional objective gait analysis trial was also performed in conjunction with the PT. Before the PT, the horse was sufficiently habituated to the task, defined as the horse walking from the point of release (see below) to the bucket without guidance from the handler. For the procedure, each horse was led by the owner or a representative for approximately 5 meters before being released and allowed to walk freely to a bucket of feed placed clearly visible on a straight line from at approximately 10m distance from the point of release. The horse handler was instructed to alternate between the left and the right side when leading the horse, and to let the horse walk independently without guidance after the release. When the horse halted and lowered its head to reach the bucket, the first forelimb that was kept protracted for >2.5 seconds was recorded. To avoid disturbances, the horses were filmed from either side using wall- or fence-mounted cameras (GoPro Hero 7 Black or GoPro 3+ Silver, GoPro, San Mateo, CA, United States), and video recordings were observed after data collection. To ensure sufficient distance between the hooves, the chestnut of the forelimb farthest from the camera had to be judged as visible from a 90° angle for the observation to be valid. All valid observations were labelled as “LEFT” (if the left forelimb was protracted) or “RIGHT” (if the right forelimb was protracted). The procedure was repeated with the goal of obtaining at least 15 valid observations, based on existing literature [14, 15]. The PT was performed on a flat surface in a restricted area, in a stable or other suitable enclosure depending on the availability. Observations where the horse halted before reaching the bucket or failed to walk in a reasonably straight and non-hesitant manner (judged by the same person for all horses, either on site or afterwards with use of video recordings), were not considered valid. In cases where it was not possible to obtain enough valid observations, the horse in question was excluded from this part of the study, except for cases where a clear result could be achieved with <15 valid observations; i.e. additional observations would not influence the classification based on a binomial test, where the limit for statistical significance corresponded to 12 out of 15 observations in the same direction (see statistical analysis section; e.g. seven “LEFT” and seven “RIGHT” observations or 13 “LEFT” and no “RIGHT” observations).

### Statistical analysis

Statistical analysis was conducted in R (version 4.1.2) [18].

#### Similarity of movement asymmetry parameters between visits

In order to approximate how similar single asymmetry parameters were between visits, an intra-class correlation coefficient (ICC) was calculated from a two-way random effects model with absolute agreement and an average of *k* measurements [19] using the package “psych” [20]. An ICC was calculated for each of the asymmetry parameters, comparing the agreement with a confidence level of 95%. Only horses with no missing objective gait analysis data from visits V1-V3 (N = 46) were included in the ICC analysis.

#### Correlation between questionnaire responses and movement asymmetry

Responses from questions 2–5 (but not 2b) were converted to binary responses; if a rider had answered “LEFT” or “RIGHT”, the answer was converted to “YES”. Linear mixed models using the package “lme4” [21] tested correlation between the binary questionnaire responses (presented in Table 2) and the absolute values of asymmetry parameters (outcome variable) with regards to stride duration and surface. This was done to investigate whether the rider could perceive a sidedness (caused by an asymmetry), but not assign it to a certain side or limb. The questionnaire answers were included as fixed factors separately in each model for every asymmetry parameter. Question 1 was modeled against TAS, questions 2a and 2b were modeled against the pelvic asymmetry parameters, question 4 against the head asymmetry parameters and question 3 and 5 against all four asymmetry parameters. Surface and mean stride duration (as proxy for speed) were set as fixed factors in all models. The horses contributed with unequal amounts of observations, both when it comes to questionnaire responses and to objective gait analyses. To account for multiple observations within subject, “horse” was set as random effect in all models. Normality was scrutinized using QQ-plots and homoscedasticity was checked by plotting residuals against fitted values of the model. Response variables rendering skewed QQ-plots were transformed prior to hypothesis testing using the Box and Cox maximum likelihood-like approach from the package “car” [22] in order to achieve normality. Since the dataset was unbalanced, a type III ANOVA using Kenward-Roger approximation for degrees of freedom from the base R function anova was performed for the responses from question 1 and 2b in the questionnaire. Post hoc analyses were performed in cases where the respective ANOVA output indicated statistical significance using an F-test based on the estimated marginal means from the model, with the use of the package “emmeans” [23].

**Table 2:** Questionnaire responses.

		Answers, expre	ssed as % of	responders
Question	Preset answer options	V1	V2	V3
1. Do you perceive your horse as having a sidedness? If so, please grade it.	*No* *Mild* *Moderate* *Severe* *No perception/did not answer*	*25%* *46%* *12%* *2%* *15%*	*25%* *42%* *12%* *4%* *18%*	*20%* *63%* *15%* *2%* *0%*
2a. Do you perceive your horse as having a weaker hindlimb?	*No* *Yes (left or right)* *No perception/did not answer*	*38%* *38%* *23%*	*35%* *42%* *23%*	*29%* *66%* *5%*
2b. If so, is it most noticeable as inner or outer limb?	*Equally* *Inner* *Outer* *No perception/did not answer*	*15%* ^*a*^ *35%* ^*a*^ *50%* ^*a*^ *0%* ^*a*^	*13%* ^*a*^ *33%* ^*a*^ *54%* ^*a*^ *0%* ^*a*^	*19%* ^*a*^ *41%* ^*a*^ *37%* ^*a*^ *4%* ^*a*^
3. Which side of your horse do you perceive as the stiffest?	*None* *Left* *Right* *No perception/did not answer*	*27%* *23%* *27%* *23%*	*30%* *19%* *24%* *26%*	*39%* *29%* *29%* *2%*
4. Which side of your horses’ neck do you perceive as the stiffest?	*None* *Left* *Right* *No perception/did not answer*	*46%* *17%* *15%* *21%*	*42%* *16%* *19%* *23%*	*51%* *17%* *27%* *5%*
5. Do you perceive it as more difficult for your horse to perform canter strike offs in any lead?	*None* *Left* *Right* *No perception/did not answer*	*52%* *17%* *17%* *13%*	*61%* *12%* *11%* *16%*	*59%* *17%* *17%* *7%*
**Number of responders (denominator)**		**52**	**57**	**41**

Expressed as percentages of total number of responders for each visit, from visits V1-V3.

^a^Percentages calculated from number of responders having stated “yes” (left or right) on previous question (2a).

Secondly, parameters that differed significantly in the binary analysis were further scrutinized, now using side-specific values (i.e. not absolute values) to test whether the rider could also assign their perception of a sidedness to a certain side or limb, and if the perceived sidedness would then correspond to side-specific values of vertical asymmetries. This was done using contingency tables that were used to manually calculate sensitivity and specificity. A side-specific analysis was conducted on these questions in order to assure that the actual side of the asymmetry corresponded to the answer about rider-perceived sidedness provided by the rider. The asymmetry parameter values, classified according to the thresholds (described earlier), were tested against the untouched categorical responses of the survey using Cramer’s V (Fisher’s exact test). Significance was set to p<0.05 for all analyses.

#### Preference test

For each horse, laterality index (LI) was calculated by dividing the difference between the sums of “RIGHT” and “LEFT” observations by the sum of all observations multiplied by 100. Thus, rightward and leftward results were discriminated with positive and negative indexes, respectively. Exact binomial tests (using the base R function binom.test) were then performed for each horse to investigate whether one side was favored over the other, i.e., if the response was significantly different from a null hypothesis of 0.5, here corresponding to equal counts of “LEFT” and “RIGHT” observations. Based on outputs from the binomial tests, each horse was classed as either positive (if p<0.05) or negative (if p ≥0.05) on the preference test. Out of the horses with positive results (i.e., a preferred side, indicated by p-value <0.05 on binomial tests) on the preference test each individual was classed as either left- or right-sided depending on which side was favored over the other during the test procedure.

Correlation between asymmetry parameters (using the movement data collected in conjunction with the PT) and LI was tested by fitting linear regression models using the base R function lm. This was performed for, in turn, both absolute values and side-specific values. This approach was used to test if the specific side preference correlated to the limb to which the asymmetry was attributed (analyzed using positive and negative values of asymmetry parameters and LI respectively), or if the magnitude, and not the side (left or right limb), of the asymmetry was of importance (analyzed using absolute values of asymmetry parameters and LI). Correlation was tested using a two-sided Pearson’s r with confidence level set to 95%.

## Results

### Owner-perceived sidedness

#### Study population V1-V3

Data from a total of 65 warmblood riding horses, aged 3–5 years (mares: n = 36, geldings: n = 28, stallions: n = 1), contributing with a total of 150 objective gait analysis trials, were included in V1-V3 for this study. Time between 2 consecutive visits ranged between 56–196 days, with a mean of 101 days, for included horses. Furthermore, a total of 135 questionnaires were collected in conjunction with V1-V3. Each horse, on average, contributed with 2.1 questionnaires and 2.3 objective gait analysis trials which were included in statistical analysis; 10% of the data points were missing before analysis, and 17% were omitted due to the exclusion criteria.

Questionnaire responses from V1-V3 on the questions used in this study are presented in Table 2.

Descriptive asymmetry data from V1-V3 are time standardized and presented in Table 3. For these visits, 60% at V1, 70% at V2 and 60% of the horses at V3, showed at least one parameter exceeding the asymmetry threshold value.

**Table 3:** Descriptive asymmetry data for visits V1-V3.

* *	*Visit 1 (V1) *			*Visit 2 (V2) *			*Visit 3 (V3) *		
* *	*n*	*Mean ± Std* ^a^	*Range*	*n *	*Mean ± Std* ^a^	*Range*	*n *	*Mean ± Std* ^a^	*Range*
***HD***_***min***_ ***R ***	18	5.05 ± 5.60	0.49–23.41	26	7.72 ± 5.45	0.23–21.97	24	7.69 ± 5.29	0.45–20.17
***HD***_***min***_ ***L ***	34	8.12 ± 9.30	0.23–31.46	31	5.72 ± 5.39	0.25–20.08	17	7.47 ± 8.49	0.15–36.81
***HD***_***max***_ ***R ***	21	6.66 ± 4.79	0.11–19.03	29	6.88 ± 5.50	0.43–21.40	20	6.21 ± 5.03	0.02–18.10
***HD***_***max***_ ***L ***	31	5.75 ± 5.54	0.12–21.81	28	5.29 ± 4.33	0.17–16.72	21	7.57 ± 5.41	0.08–19.30
***PD***_***min***_ ***R ***	31	3.07 ± 1.74	0.30–6.87	30	3.28 ± 2.49	0.21–7.27	24	3.17 ± 2.67	0.17–9.01
***PD***_***min***_ ***L ***	21	2.44 ± 2.71	0.02–10.76	27	2.43 ± 1.66	0.51–6.23	17	2.11 ± 1.85	0.05–5.49
***PD***_***max***_ ***R ***	29	2.35 ± 1.82	0.02–7.94	28	2.90 ± 2.66	0.01–9.98	26	2.44 ± 1.74	0.08–6.83
***PD***_***max***_ ***L ***	23	2.41 ± 2.13	0.08–8.64	29	2.49 ± 2.16	0.02–8.42	15	2.28 ± 2.01	0.39–6.82
***TAS***	52	11.74 ± 5.91	3.33–24.84	57	11.89 ± 5.27	2.88–26.18	41	12.32 ± 6.21	2.25–35.38

Means are calculated from all horses in each visit, i.e. not only horses exceeding asymmetry thresholds. The number of horses exceeding each asymmetry threshold is given in “n” column. In the bottom row, total number of included horses is given for each visit. Data are averaged over horses and the four asymmetry parameters (HDmin, HDmax, PDmin, PDmax), as well as the total asymmetry score (TAS). For the asymmetry parameters, left (L) or right (R) side is specified and values are given in the unit of corrected millimeters. Absolute values are used.

^a^Standard deviation

#### Agreement between visits

Agreement of asymmetry between visits (V1-V3) was good (ICC ranging between 0.77–0.815, p<0.0001) for all parameters except for HDmin where the agreement between trials was poor (ICC = 0.39, p<0.05) indicating a higher variation between visits in this variable.

#### Association between asymmetry parameters and questionnaire responses

**Fig 1:**
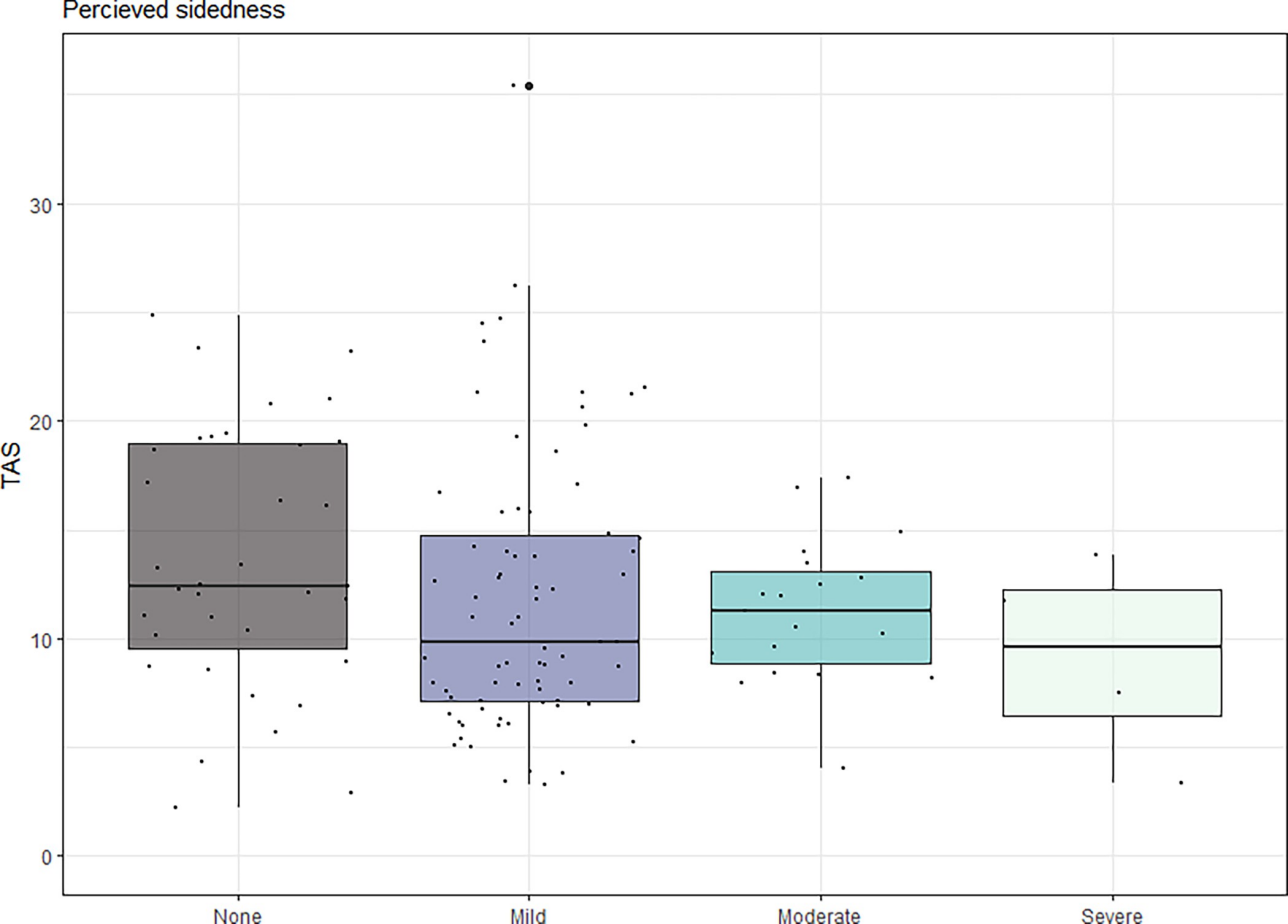
Total asymmetry score (TAS) plotted against groups of rider-perceived sidedness. Based on answers to questionnaire question 1, see Table 2. Each data point is plotted individually jittered over the boxplot. Upper and lower part of boxes correspond to the first and third quartiles and the line corresponds to the median value. Whiskers extend to the minimum and maximum value but no longer than 1.5 times the interquartile range. Values outside the whiskers are presented individually as larger dots.

**Fig 2:**
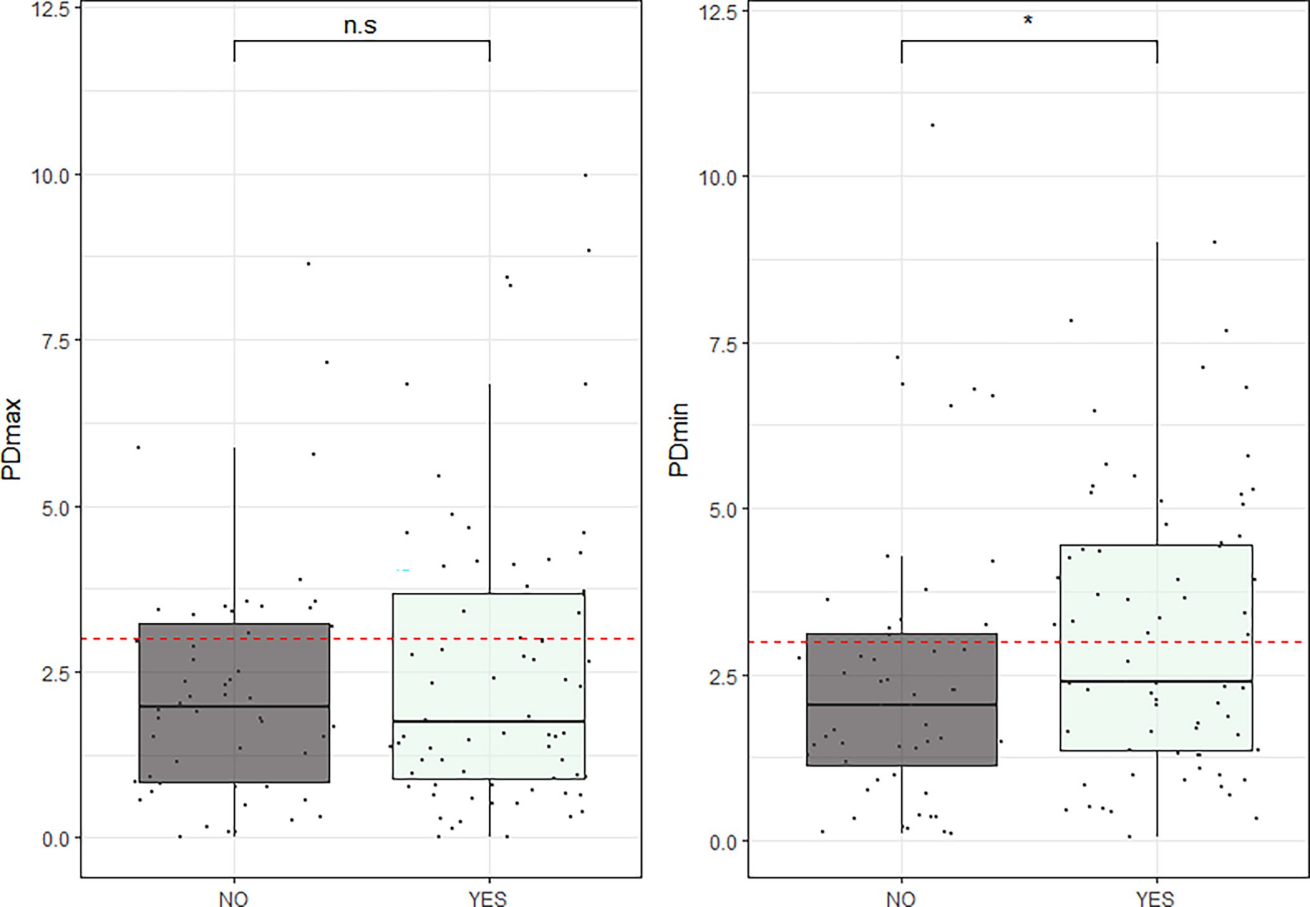
Boxplot of absolute values of hind limb asymmetry plotted against questionnaire responses. Responses are converted to binary YES/NO (LEFT and RIGHT converted to YES) based on questionnaire answers for question 2a (see Table 1). Each data point is plotted individually jittered over the boxplot. Upper and lower part of boxes correspond to the first and third quartiles and the line corresponds to the median value. Whiskers extend to the minimum and maximum value but no longer than 1.5 times the interquartile range. Dotted red line marks the value of 3 mm. N.s. = not significant. Statistical significance (p<0.05) is indicated by *.

There was no difference in TAS between groups of graded rider-perceived sidedness according to the ANOVA (p = 0.585), see Fig 1. Estimates of the mixed model contrasts of the absolute values of vertical asymmetry parameters tested against the binary questionnaire answers (i.e. LEFT and RIGHT converted to YES) are presented in Table 4. Statistically significant associations between asymmetry parameters and questionnaire responses were not seen for any of the questionnaire questions except for question 2a (perceived weaker hind limb), where a weak effect on PDmin (p = 0.023) was seen, see Table 4 and Fig 2. Most of the variation in the data was explained by the random effects (horse) or fixed effects (stride duration) but despite that, significantly higher PDmin values were seen among the horses where the owners reported a weaker hindlimb. However, a positive questionnaire answer was still a weak predictor for a high PDmin with a sensitivity of 32% and a specificity of 91%.

**Table 4:** Contrasts between estimated marginal means for questionnaire responses.

Independent variable	Dependent variable	Estimated contrast (between YES and NO)	SE	p-value
**PDmax **	Weak hind limb (2a)	0.113	0.084	0.182
**PDmin **	Weak hind limb (2a)	0.222	0.096	0.023 *
**HDmax **	Stiff side (3)	0.291	0.154	0.061
**HDmin **	Stiff side (3)	0.088	0.071	0.223
**PDmax **	Stiff side (3)	0.050	0.094	0.597
**PDmin **	Stiff side (3)	0.030	0.096	0.755
**HDmax **	Stiff neck (4)	0.247	0.141	0.083
**HDmin **	Stiff neck (4)	0.061	0.068	0.371
**HDmax **	Lead canter (5)	0.040	0.152	0.793
**HDmin **	Lead canter (5)	0.111	0.073	0.128
**PDmax **	Lead canter (5)	0.082	0.080	0.297
**PDmin **	Lead canter (5)	0.090	0.110	0.413

Responses are converted to binary YES/NO (where LEFT and RIGHT are converted to YES). SE = standard error. The question number from Table 1 used as dependent variable is stated in brackets. Contrasts are presented on the transformed scale. P-values <0.05 are indicated by *.

**Fig 3:**
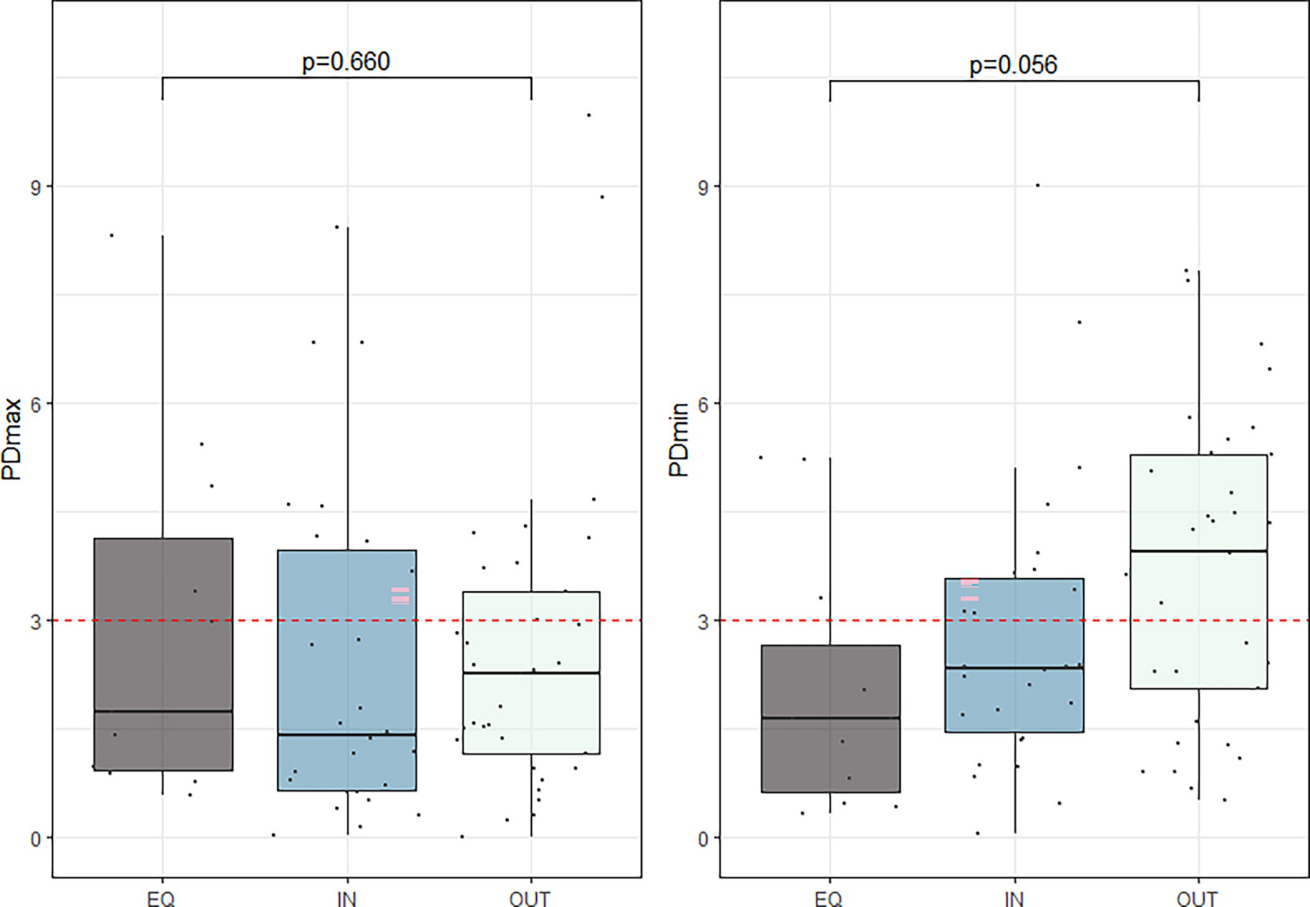
Boxplot of absolute values of hind limb asymmetry plotted against questionnaire responses from question 2b. See Table 1. Each data point is plotted individually jittered over the boxplot. Upper and lower part of boxes correspond to the first and third quartiles and the line corresponds to the median value. Whiskers extend to the minimum and maximum value but no longer than 1.5 times the interquartile range. No significant difference in TAS was seen between these groups. Dotted red line marks the value of 3 mm. P-values from ANOVA is presented in the plots.

There was a moderate, but not significant correlation between which hind limb (left or right) the owner perceived as weaker and any of parameters PDmin or PDmax exceeding the threshold for the specific limb (V = 0.2732, p = 0.06). In general, owners tended to report the presence of a weaker hind limb more often than a vertical asymmetry was detected. The correlation between question 2b (most noticeable as inner or outer limb on the circle) and the absolute values of the pelvic asymmetry variables are presented in Fig 3. No significant difference between groups could be found.

#### Preference tests

A total of 40 horses were subjected to the PT and corresponding objective gait analysis. The ages of horses included in this subgroup ranged between 4–7 years when this procedure was executed. Eight horses were excluded due to failure in providing enough valid observations in the PT, and one was excluded due to loss of video. Thus, 32 horses were finally included in this part of the study. In five cases, horses with only 13 (1 case) or 14 (4 cases) valid observations were included because of obvious outcome even without presence of the last observation(s) (six versus seven, six versus eight, or seven versus seven left/right observations, or vice versa). Descriptive statistics are presented in Table 5. Seven out of the 32 included horses (22%) received a p-value <0.05 on the binomial test (corresponding with ≥12/15 observations attributed to one side) and thus showed a positive result in the PT. Two and five of the horses presented a left- and right-sided preference, respectively. The TAS was similar between groups.

**Table 5:** Descriptive results from PT and objective gait analysis.

Preference test	n	Mean LI	Std^a^	Mean TAS (|mm|)	Std^a^
**Positive**	7	87.46	14.98	10.47	1.40
***Right sided***	5	95.15	6.65	10.66	1.29
***Left sided***	2	68.24	2.12	9.99	2.12
**Negative**	25	19.63	15.17	12.60	6.69

Means and standard deviations of laterality index (LI) and total asymmetry score (TAS, calculated from the objective gait analysis performed in conjunction with PT) of horses with positive and negative (also divided by left and right directions) results from the preference tests, respectively, are given. Absolute values of laterality indexes are used. ^a^Standard deviation.

**Fig 4:**
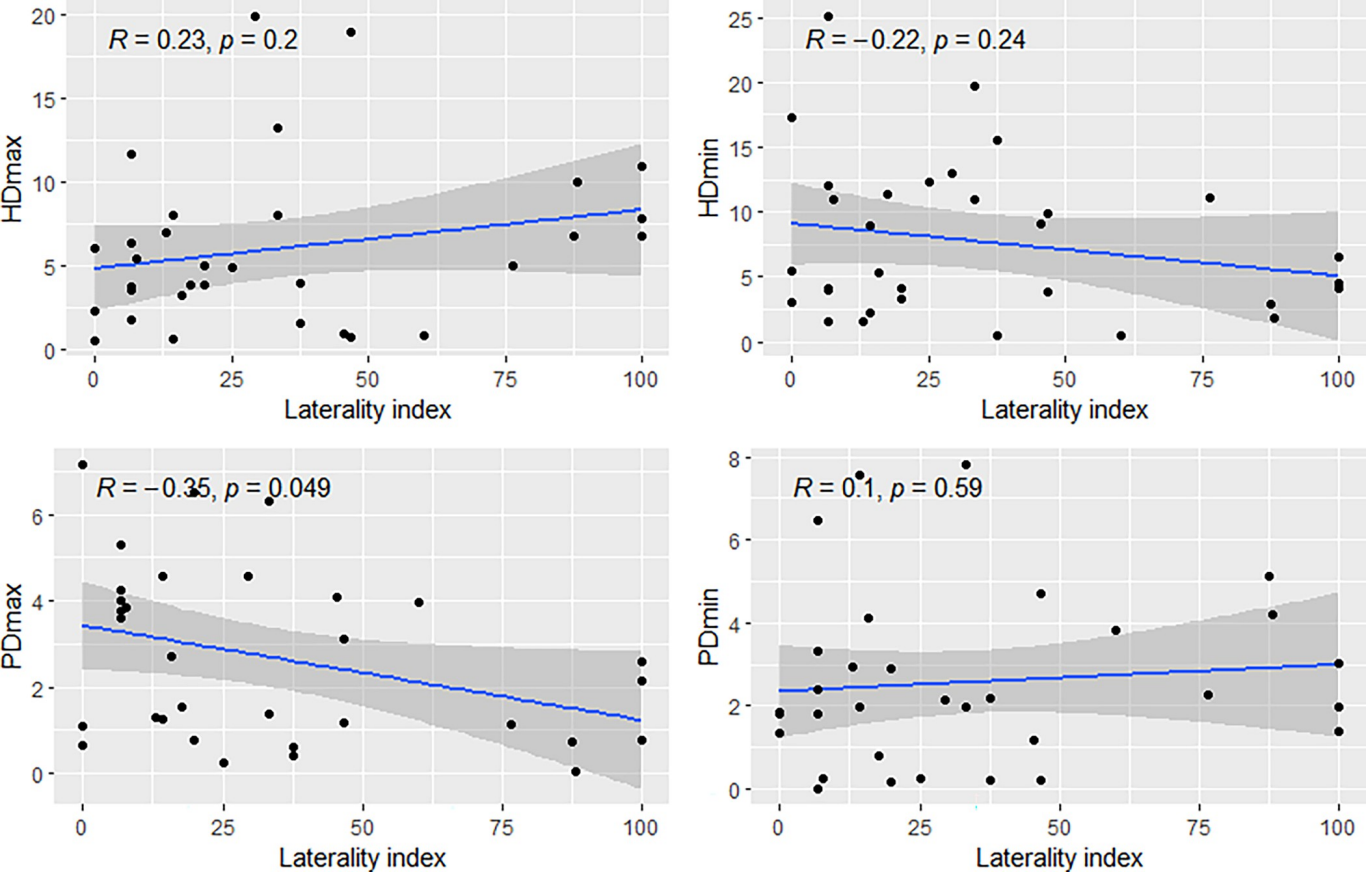
Linear correlation between asymmetry parameters and laterality index, for the asymmetry parameters HDmax, HDmin, PDmax and PDmin. Asymmetry parameters are presented on the y-axes and laterality index on the x-axes. Absolute values are used for both asymmetry parameters and laterality index. R-values are stated indicating the degree of correlation. P-values indicate level of significance.

The percentage of horses with at least one vertical displacement parameter exceeding thresholds was 59% while the percentage of horses exerting a side preference in the PT was 22%. No correlation between absolute values of LI and TAS could be found (R = -0.098, p = 0.60). A statistically significant negative correlation was seen for the degree of laterality when plotted against absolute values of PDmax (displayed in Fig 4). This correlation was not significantly different from zero within any of the asymmetry parameters when taking left and right side into account for both LI and asymmetry parameters.

## Discussion

In this study, correlations between vertical asymmetries and measures of motor laterality in young, “owner-sound” warmblood horses are investigated. To our knowledge, this is the first study investigating associations between vertical asymmetries and motor laterality as well as rider-perceived sidedness.

We demonstrated several challenges with investigating the relationship between motor laterality and vertical asymmetries in horses. First, rider perceived sidedness was assessed in V1-V3 with the use of a questionnaire. The questions used here are thought to reflect perceptions of sidedness that riders commonly claim to experience, and the goal was to investigate whether these perceptions could be associated with vertical asymmetries. On the questionnaires associated with V1-V3, a majority of responders reported a perceived sidedness in their horses in five out of six questions (V1 and V2) and four out of six questions (V3). However, no clear left- or rightward bias could be seen in the responses. A statistically significant correlation between vertical asymmetries and rider-perceived sidedness could only be seen for one of the questionnaire questions and one asymmetry parameter (perception of a weaker hindlimb and higher magnitude of PDmin in a hindlimb), and this was only seen when the directions of asymmetry and perceived sidedness were not taken into account. Thus, this result is, although significant, most likely of little (if any) biological importance. Thus, it seems that owner perceived sidedness as assessed by the questions used in the present study does very little to explain the prevalence of asymmetries in this population. Although a high specificity was seen for this factor, the sensitivity was low which in turn implies that the perception of a weaker hindlimb in a horse is a weak predictor for higher PDmin values. However, in clinical settings, owners may in some cases report a rider perception as the only clinical sign, and a report of a weaker hindlimb could provide some information to the clinician even though its potential value as, for instance, a screening tool is limited. However, the sensitivity may be higher in a population of lame horses.

According to the ICC analysis, asymmetry parameters generally remained consistent over the three visits. However, according to the mixed model analyses, they did not seem to be correlated to questionnaire responses. This suggests that other factors than asymmetries affect rider-perceived sidedness. In this context, it must be pointed out that it is somewhat unclear what rider-perceived sidedness represents. While it could potentially represent inherent motor laterality in horses, it could also represent a reflection of the riders’ sidedness; the rider’s perception may e.g. be influenced by his or her own laterality [13] or physical limitations, where the latter may change over time. It could also be assumed that riders may interpret behaviors from horses differently, which makes comparisons between individual horses with different riders challenging. Additionally, the level and skills (which were not standardized in this study) of the riders could potentially influence their ability to perceive sidedness in their horses, which adds further complexity to the matter. In future studies, it could therefore be of interest to include this type of data about riders in analyses.

Second, motor laterality was assessed using a preference test, where each horse’s protracted forelimb while eating from a bucket at ground level was recorded for a set of observations. In our population (consisting of a subgroup of the entire study population), 22% of horses showed a limb preference, which is similar to what was seen by Van Heel *et al*., [15], but lower than proportions reported by McGreevy *et al*., and Murphy *et al*., [12, 13]. When accounting for the directions of asymmetry and side preference, no correlation between the presence of motor laterality and vertical asymmetries could be found, and the proportion of subjects presenting with vertical asymmetries was more than two times higher than the proportion exhibiting a side preference in the PT. This implies that at least a substantial proportion of vertical asymmetries are caused by other factors than inherent motor laterality. However, no “gold standard” tests for evaluation of motor laterality in horses are described in the current literature, which makes comparisons between different studies difficult. Furthermore, it is not certain whether the PT used here accurately describes the occurrence of motor laterality in our population. As our test only investigates protraction of forelimbs, it could, for instance, be hypothesized that it is less accurate for detection of side preference in hind limbs. It is also a possibility that factors such as conformation traits (such as e.g. shape of hoofs) and sensory input (such as e.g. noises from the stables that could not be eliminated) influenced results. Measures were taken to minimize sensory input, but these are regardless difficult to control entirely. Some earlier studies have used multiple measures of laterality [15, 24], which could potentially help in obtaining a better understanding of an individual’s side preference.

This study is also, to our knowledge, the first to describe the prevalence of asymmetries in 3–5-year-old riding horses. A large proportion of our subjects exhibited vertical asymmetries above previously described threshold levels, which is in line with previous research [3–6]. To this date, very little is known about the development of these asymmetries. In this study, we are able to draw some conclusions regarding the changes of these asymmetries over time. According to the ICC analyses, three out of four asymmetry parameters showed good agreement when comparing them between visits, suggesting that asymmetry parameters did not change substantially between these time points, except for HDmin, where the agreement was poor. A higher variation between visits (which was seen for HDmin) would suggest more short-term causes, such as e.g. recent shoeing or acute injuries, while low variation (which was seen for HDmax, PDmin, and PDmax) instead suggests that these asymmetries remain somewhat stable over time. This, in turn, implies more persistent or congenital causes such as, for instance, inherent motor laterality or conformation. Still, causes such as e.g. chronic pain due to, for instant, low-grade joint inflammation still cannot be completely ruled out. No horses with obvious deviations from normal conformation are included in the study, and all horses were clinically examined. However, no standardized conformation measurements or detailed diagnostic procedures (such as e.g. radiologic exams) were performed, which could have provided more information.

Lastly, it is important to point out that the relatively large amount of missing data, which is partly due to the nature of the investigation (ongoing over a relatively long time period). Furthermore, a substantial amount of data were excluded due to clinical findings which could be signs of locomotor pathology. One of the purposes of this research was to study horses that would generally be considered as sound, which is why it was considered necessary to exclude horses with clinical findings that might indicate orthopedic disease. However, determining in what state a horse should be to be considered as sound represents a challenge and this perception is likely to differ between different assessors.

We also recognize that inclusion of subjects may to some extent be influenced by the conditions under which horses were evaluated. Due to the fact that a large proportion of the movement data were collected under field conditions, it was not possible to evaluate the trot on more than one surface type. When it comes to objective data, this was accounted for in the statistical analysis, where surface type was included as a factor in the mixed models analyses. However, the surface type may to some extent have influenced the subjective assessments, which represents a limitation as subjectively graded lameness was an exclusion criterion.

## Conclusion

In this study, which is a first attempt to investigate relations between inherent motor laterality and vertical asymmetries, no convincing evidence to support a correlation between rider-perceived sidedness and vertical asymmetries was obtained. It remains unclear why such large proportions of “owner-sound” horses exhibit vertical asymmetries. This may be due to usage of improper methods to measure laterality. Thus, motor laterality cannot, based on our results, be ruled out as a contributing factor to vertical asymmetries. Further studies involving larger numbers of subjects, as well as more measures of laterality, are warranted to investigate whether some fraction of vertical asymmetries seen in horses could be explained by inherent motor laterality.

## Supporting information

S1 Text Pdf file containing the questionnaire about rider-perceived sidedness used in V1-V3. (PDF)

S1 Table Microsoft excel file containing data used in statistical analyses. (XLSX)

S2 Table Microsoft excel file containing data used in statistical analyses. (XLSX)
