# Supplementary material for: Perceived sidedness and correlation to vertical movement asymmetries in young warmblood horses
Source: PLoS One. 2023 Jul 7;18(7):e0288043. doi: 10.1371/journal.pone.0288043 (PMC10328353; doi:10.1371/journal.pone.0288043)
Supplement: S1 Text — (PDF) [file pone.0288043.s001.pdf]

S1 text

Note: the answer option “no perception” was also included for all questions.

Do you perceive your horse as having a sidedness? If so, please grade it.

☐ No

☐ Yes, mild

☐ Yes, moderate

☐ Yes, severe

Do you perceive your horse as having a weaker hindlimb?

☐ No ☐ Yes, left ☐ Yes, right

If so, is it most noticeable as inner or outer limb?

☐ Inner limb

☐ Outer limb

☐ Equally

What gives you the perception of one hindlimb being weaker than its counterpart?

__________(Free text answer)___________

Which side of your horse do you perceive as the stiffest when trying to obtain a curvature?

☐ None ☐ Left ☐ Right

Which side of your horses’ neck do you perceive as the stiffest?

☐ None ☐ Left ☐ Right

Does your horse ”float out” on the circle in any direction?

☐ No ☐ Yes, on the left hand ☐ Yes, on the right hand

Does your horse ”push inwards” on the circle in any direction?

☐ No ☐ Yes, on the left hand ☐ Yes, on the right hand

On which rein does the horse take more support?

☐ Equal on both reins ☐ Left rein ☐ Right rein

Do you perceive it as more difficult for your horse to perform canter strike offs in any direction?

☐ No ☐ Yes, for gallop on the left leg ☐ Yes, for gallop on the right leg

Is it more difficult to perform changes of leg in any direction?

☐ No ☐ Yes, change of leg from left to right ☐ Yes, change of leg from right to left

Does your horse push its shoulder outwards? If so, which shoulder and what direction?

☐ Right shoulder, right hand ☐ Right shoulder, left hand

☐ Left shoulder, right hand ☐ Left shoulder, left hand

☐ No

Is it more difficult for your horse to move off any of the legs (leg yielding)?

☐ No ☐ Left ☐ Right

Trot

Does your horse track up in the trot/ Does your horse place its hindlimbs on the same track as its forelimbs?

Right hand

☐ Yes, the horse is tracking up ☐ No, the hindlimbs are placed to the left

☐ No, the hindlimbs are placed to the right

Left hand

☐ Yes, the horse is tracking up

☐ No, the hindlimbs are placed to the left

☐ No, the hindlimbs are placed to the right

Do you perceive the horse to be straighter in any direction?

☐ No, both hands are equal

☐ Yes, it is straighter on the left hand

☐ Yes, it is straighter on the right hand

Do you perceive your horse to be ”folding” any of its sides?

☐ No, both hands are equal

☐ Yes, the right side on the right hand

☐ Yes, the right side on the left hand

☐ Yes, the left side on the right hand

☐ Yes, the left side on the left hand

How do you perceive the contact between your hand and the horse's mouth in different directions in trot?

Right hand:

☐ Equal contact from both reins ☐ More contact from the right rein

☐ More contact from the left rein

Left hand:

☐ Equal contact from both reins ☐ More contact from the right rein

☐ More contact from the left rein

Canter

Does your horse have difficulties cantering in any of the leads?

☐ No, both leads are equal ☐ Yes, gallop of the left leg is more difficult

☐ Yes, gallop of the right leg is more difficult

Does your horse track up in the canter/ Does your horse place its hindlimbs on the same track as its forelimbs?

Right hand

☐ Yes, the horse is tracking up ☐ No, the hindlimbs are placed to the left

☐ No, the hindlimbs are placed to the right

Left hand

☐ Yes, the horse is tracking up ☐ No, the hindlimbs are placed to the left

☐ No, the hindlimbs are placed to the right

How do you perceive the contact between your hand and the horse's mouth in different directions in the canter?

Right hand:

☐ Equal contact from both reins ☐ More contact from the right rein

☐ More contact from the left rein

Left hand:

☐ Equal contact from both reins ☐ More contact from the right rein

☐ More contact from the left rein
